# The Battle Against COVID-19 in Jordan: From Extreme Victory to Extreme Burden

**DOI:** 10.3389/fpubh.2020.634022

**Published:** 2021-01-27

**Authors:** Moawiah Khatatbeh

**Affiliations:** Department of Basic Medical Sciences, Faculty of Medicine, Yarmouk University, Irbid, Jordan

**Keywords:** COVID-19, Jordan, lockdown, middle east, surveillance, policy

## Introduction

Since the emergence of COVID-19 in Wuhan, China, massive public health interventions have been implemented to contain the outbreak (1). However, within a few weeks, the virus had spread rapidly throughout China and within 1 month to several other countries (2). As of November 25th, 2020, worldwide, more than 60 million people were confirmed to have the novel coronavirus, with more than 1.42 million deaths (3). In Jordan, the first case of COVID-19 was discovered on March 2nd, 2020 when the government implemented an immediate and large range of non-pharmaceutical interventions to combat the spread of the virus (4). Patients were immediately and completely isolated in specialized hospitals, and quarantine was initiated as per official guidelines to medical professionals and using all personal protective equipment (5). These interventions were empowered by the activation of the National Defense Law on March 17th, 2020. The activation of the law led to the suspension of studies at educational institutions, closure of borders, stopping prayers in places of worship, and banning all large gatherings.

## The Epidemiological Situation During the Lockdown

Three weeks after the discovery of the 1st case in Jordan, the total number of cases in the country was 98. This number of cases constituted a red flag and pushed the government toward implementing more strict interventions including complete nationwide lockdown for 3 days (March 22–24) (6). As of July 2nd, 2020 (4 months after the 1st confirmed case), the total number of cases of COVID-19 in the country was 1,136 (6).

During the 5th month of the epidemic in Jordan (from July 2nd to August 2nd 2020), only 77 new cases were diagnosed heading the total number of cases to 1,213 and 11 deaths, despite the available and convenient diagnostic testing (6). Simultaneously, Jordan had the lowest number of COVID-19 cases compared with other countries in the Middle East despite having had the disease before some countries such as Turkey and Bahrain (4).

Early strict intervention measures showed evidence of containing and suppressing the disease and resulted in a low incidence. Among all countries in the Middle East, Jordan was, in principle, in a better position to fight against the disease (7, 8).

Over the course of the COVID-19 pandemic, lockdown measures have been shown to affect human psychology and the economy. It has been reported that 8.0% of individuals had stress, while the prevalence of depression ranged from 16 to 28% (9). Furthermore, in almost 90% of the world, lockdown measures led to people not going out to markets or restaurants; workplaces have been closed, flights have been banned, and school has been suspended (9), leading to an unprecedented economic disaster (10). The well-controlled epidemiological situation led to a feeling, among both the public and decision makers, that the epidemiological situation was well-controlled and acceptable, which encouraged local authorities to loosen lockdown measures to prevent further deterioration of the economy and maintain public mental health (11).

## Consequences of Easing the Lockdown Measures

One month after relaxing the lockdown measures (from August 2nd to September 2nd, 2020), the number of cases had almost doubled, bringing the total to 2,161. In the following month, (from September 2nd to October 2nd, 2020), the situation became uncontrollable, and the epidemiological investigation became impossible after reaching 13,650 cases (6).

As of November 26th, 2020, the total number of COVID-19 cases in Jordan had reached more than 200,000 (6). The disquieting news is that daily testing trends in the last 14 days show that the positivity rate has reached 28.8% with a 7-days moving average of 20.46%. Furthermore, the number of deaths of COVID-19 cases in Jordan is distressing. The case fatality rate reached 1.23%, which is higher than in other countries in the Middle East such as Qatar, 0.17%; United Arab Emirates, 0.35%; and Bahrain, 0.40% (3).

## Discussion

Alleviating lockdown measures, especially at the Syrian borders, as stated by the Minister of Health, has led to this unfortunate situation, which has been aggravated by having a high prevalence of chronic non-communicable diseases among the Jordanian population (12), which places these vulnerable groups at higher risk for catching the COVID-19 virus.

Statistics in Table 1 are predictive of an increase in the total number of cases and the fatality rate in Jordan to an unmanageable level, resulting from a lack of resources, low economic status, and the high prevalence of chronic diseases among the population. Furthermore, despite being high, the 7-days moving average of positivity rates in the Jordanian population does not reflect the actual prevalence of COVID-19 in the country for many reasons. First, only those who suspect that they have the virus as a result of becoming symptomatic or having contact with confirmed cases take the test. Second, the aforementioned rate is not a result of screening procedure but a result of testing the high risk groups. Third, not all high risk individuals take the test. Finally, in the era of COVID-19, test sensitivity and specificity issues remain a concern (13).

**Table 1:** COVID-19 daily testing and fatality trends in Jordan in the last 14 days.

**Date**	Number of Tests^*^	Number of cases^*^(positivity rate)	Number of deaths^*^
25 Nov	27,098	5,025 (18.5%)	62
24 Nov	24.664	4.586 (18.6%)	78
23 Nov	25,349	4,981 (19.6%)	66
22 Nov	23,953	5,268 (22.0%)	64
21 Nov	18,518	3,826 (20.6%)	56
20 Nov	22,814	4,940 (21.6%)	63
19 Nov	24,210	5,470 (22.6%)	84
18 Nov	30,792	7,933 (25.8%)	60
17 Nov	27,441	6,454 (23.5%)	66
16 Nov	26,475	5,861 (22.1%)	71
15 Nov	9,583	2,373 (24.8%)	68
14 Nov	16,502	4750 (28.8%)	86
13 Nov	16,771	4,469 (26.6%)	71
12 Nov	24,128	5,685 (23.6%)	80
7-days moving average of positivity rate			20.46%
Total case Fatality rate as of -November 25th, 2020			1.23%

* *From: https://corona.moh.gov.jo/en/mediacenter/*.

In Jordan, as a tribal community, these figures are projected to increase in the next few weeks after the excessive number of illegal public gatherings recently, on Election Day (10th November, 2020) and the following days. Over those days, adherence to protective measures was non-existent, and violating the laws and regulations of physical distancing was the predominant feature of those gatherings. Decision makers in Jordan had to think carefully before easing the lockdown measures and pay attention to the consequences of public gatherings. They should have learned from previous experiences, such as when a wedding ceremony led to a large outbreak of COVID-19 in northern Jordan in the early days of the epidemic (14).

Easing lockdown measures included travelers' self-managed quarantines rather than government-managed quarantine policy, which was adopted in New Zealand and proved to be successful in combating the spread of the pandemic (15). Moreover, Ethiopia has succeeded in preventing the spread of the virus since it postponed the national elections and implemented complete transport lockdown (16). Several countries, including Jordan, achieved control over the disease by combining moderate physical distancing measures with self-isolation and contact tracing (17). Contact tracing is plausible at a low rate of infection. However, a large number of contacts would need to be traced and tested if the incidence of symptomatic cases is high. In fact, this scenario has occurred in most countries, including Jordan. In such a scenario, the best approach is to maintain closure of borders, isolation or quarantine, physical distancing, school closures, remote working, and contact tracing, with a view to keeping infection rates low (17). Once the incidence of infection goes higher, countries and health authorities would be unable to trace contacts, and all other interventions would become meaningless.

Jordan's experience in dealing with the COVID-19 pandemic is an example that can be of great benefit to other countries that have achieved control of the virus, so that they can avoid any serious mistakes. Moreover, great lessons can be learned from such a scenario, as to how to best counter similar threats in the future. Meanwhile, more strict regulations on social distancing, further education of the public about the importance of protective measures, application of severe penalties for violators, and the encouragement of good nutrition, especially for older adults, are the sole means to decrease the incidence of COVID-19.

## Author's Note

During the COVID-19 pandemic, countries have responded in diverse ways to combat its spread. In the first 4 months of the epidemic, Jordan implemented strict measures to fight the disease, which proved to be effective in decreasing infection rates in the country. However, easing these measures has its price. Jordan now has an immense number of cases *per capita*, and these numbers are projected to increase in the coming weeks. Jordan's experience in dealing with the COVID-19 pandemic is an example that can be of great benefit to other countries that have achieved control of the virus, so that they can avoid any serious mistakes.

## Author Contributions

The author confirms being the sole contributor of this work and has approved it for publication.

## Conflict of Interest

The author declares that the research was conducted in the absence of any commercial or financial relationships that could be construed as a potential conflict of interest.
